# Novel Oral Anticoagulants Versus Antiplatelet Therapy in Post-TAVR Patients: A Single-Center Retrospective Study

**DOI:** 10.3390/jcm14134690

**Published:** 2025-07-02

**Authors:** Ricardo A. Rodriguez Mejia, Eric Acker, Vinh Dao, Humza Rana

**Affiliations:** 1 Department of Hospital Medicine, Cape Fear Valley Medical Center, Fayetteville, NC 28304, USA; 2 Department of Internal Medicine, Cape Fear Valley Medical Center, Fayetteville, NC 28304, USA

**Keywords:** transcatheter aortic valve replacement, anticoagulation, Vitamin K antagonists, novel oral anticoagulants, antiplatelet therapy, clinical outcomes

## Abstract

**Background**: The optimal antithrombotic therapy after transcatheter aortic valve replacement (TAVR) remains uncertain. Limited data exist comparing novel oral anticoagulants (NOACs) with standard antiplatelet therapy in this population. **Methods**: We conducted a retrospective analysis of 171 patients who underwent TAVR between January 2018 and August 2024. Patients were categorized according to the discharge antithrombotic regimen as follows: NOACs (n = 27, 16%), vitamin K antagonists (VKAs; n = 8, 5%), and antiplatelet therapy only (APT-only; aspirin and/or clopidogrel without oral anticoagulation; n = 136, 79%). Due to the small VKA sample size, the primary analysis compared NOACs with APT-only. VKA outcomes were reported descriptively without statistical comparisons. **Results**: Compared with APT-only, NOAC users had significantly higher 30-day mortality (33% vs. 12%, *p* = 0.017) and 1-year mortality (41% vs. 20%, *p* = 0.048). NOACs were associated with higher rates of major adverse cardiovascular events (MACCE) at 30 days (22% vs. 8%, *p* = 0.051) and 1 year (34% vs. 17%, *p* < 0.001). After inverse probability treatment weighting, NOACs showed increased odds of 30-day MACCE (OR 5.59, 95% CI 2.56–12.18, *p* < 0.001) and increased hazard of 1-year mortality (HR 2.22, 95% CI 1.22–4.03, *p* = 0.009). **Conclusions**: NOAC use was associated with inferior outcomes compared to antiplatelet therapy in post-TAVR patients, although residual confounding cannot be excluded. Given the limited sample size and retrospective design, these hypothesis-generating findings require validation in larger prospective studies before they can influence clinical practice.

## 1. Introduction

Transcatheter aortic valve replacement (TAVR) is an expanding intervention for the treatment of aortic stenosis. TAVR has been shown to reduce mortality by 45% in early aortic stenosis compared with conservative treatments [1]. This approach is more frequently utilized than surgical aortic valve replacement (SAVR) because TAVR offers a non-inferior alternative to SAVR [2,3]. Prior to TAVR implementation, patients with advanced age, prior cardiac surgeries, or those considered higher risk may not have been considered candidates for SAVR; however, these patients may now be managed with TAVR.

The American College of Cardiology/American Heart Association (ACC/AHA) guidelines provide age-based recommendations for valve selection: for patients < 50 years without contraindication to anticoagulation, a mechanical prosthesis is reasonable (Class 2a); for patients 50–65 years, the choice should be individualized between mechanical and bioprosthetic valves with shared decision-making (Class 2a); and for patients > 65 years, a bioprosthesis is reasonable over a mechanical valve (Class 2a) [4]. However, antiplatelet and anticoagulation recommendations after these procedures are less well established.

Emerging trials, such as the GALILEO trial have indicated the use of the anticoagulant rivaroxaban and aspirin (ASA) [5]; however, these medications are associated with higher mortality rates and thromboembolic and major bleeding events. Recommendations that garner a level 2 recommendation by the 2020 ACC/AHA valvular disease guidelines include the use of ASA (between 75 mg and 100 mg daily), the use of Vitamin K antagonists (VKAs) in patients with low bleeding risk with a goal international normalized ratio (INR) of 2.5 for 3–6 months, or the use of dual antiplatelet therapy over VKA in patients who experience a thromboembolic event post bioprosthetic valve [4].

Current guidelines for anticoagulation and antiplatelet regimens are based on expert consensus supported by clinical trial data. Regarding antiplatelet regimens, most guidelines are based on extrapolated data from coronary stents [6], and research in this field is in its infancy or has yet to yield a definitive answer. In fact, the recent ATLANTIS trial, which compared apixaban with standard-of-care (warfarin or antiplatelet therapy), found that apixaban was not superior to the current standard-of-care [7]. There is also a need to establish data-driven guidelines for post-TAVR care, as each of these medications comes with its own added risk, and assessing their efficacy in this patient population is imperative. Therefore, further evaluation of existing data and clinical trials is needed. 

The optimal antithrombotic therapy following TAVR remains a topic of ongoing debate. While the benefits of anticoagulation in preventing thromboembolic events are well established, the ideal agent and duration of therapy remain unclear. Recent studies have explored various antithrombotic strategies, including single antiplatelet therapy (SAPT), dual antiplatelet therapy (DAPT), and oral anticoagulation with either VKAs or novel oral anticoagulants (NOACs) [8].

The POPular TAVI trial provided evidence that aspirin alone may be preferable to DAPT in patients without an indication for oral anticoagulation, showing a reduction in bleeding events without an increase in thromboembolic complications [9]. However, the choice between VKAs and NOACs remains controversial for patients with indications for anticoagulation, such as atrial fibrillation. Conversely, the ENVISAGE-TAVI AF trial demonstrated the non-inferiority of edoxaban to that of VKAs for net adverse clinical events, albeit with an increased risk of major bleeding [10].

Recent meta-analyses have attempted to synthesize this evidence. A comprehensive review by Malik et al. (2023) concluded that, while NOACs appeared to have similar efficacy to VKAs in preventing thromboembolic events, they were associated with a higher risk of major bleeding in TAVR patients [11]. This finding underscores the complexity of balancing thrombotic and hemorrhagic risks in this unique patient cohort. Furthermore, emerging evidence suggests that leaflet thrombosis, a potential complication post-TAVR, may be influenced by the choice of antithrombotic therapy, adding another layer of complexity to treatment decisions [12].

The heterogeneity of TAVR patients, including variations in age, comorbidities, and indications for anticoagulation, further complicates the development of standardized antithrombotic protocols. Recent guidelines from the European Society of Cardiology (ESC) and the European Association for Cardio-Thoracic Surgery (EACTS) highlight the need for individualized approaches, taking into account patient-specific factors and procedural characteristics [13]. However, these recommendations are largely based on expert consensus, emphasizing the urgent need for more robust and long-term data to guide clinical decision-making.

The goal of this study was to determine whether NOACs provide a more favorable safety and efficacy profile than VKAs in post-TAVR patients, with the expectation that NOACs are associated with lower rates of major bleeding and overall mortality while maintaining or improving rates of major adverse cardiovascular events (MACCE) and non-major adverse cardiovascular events (NACE).

## 2. Methods

### 2.1. Study Design and Population

**Figure 1:**
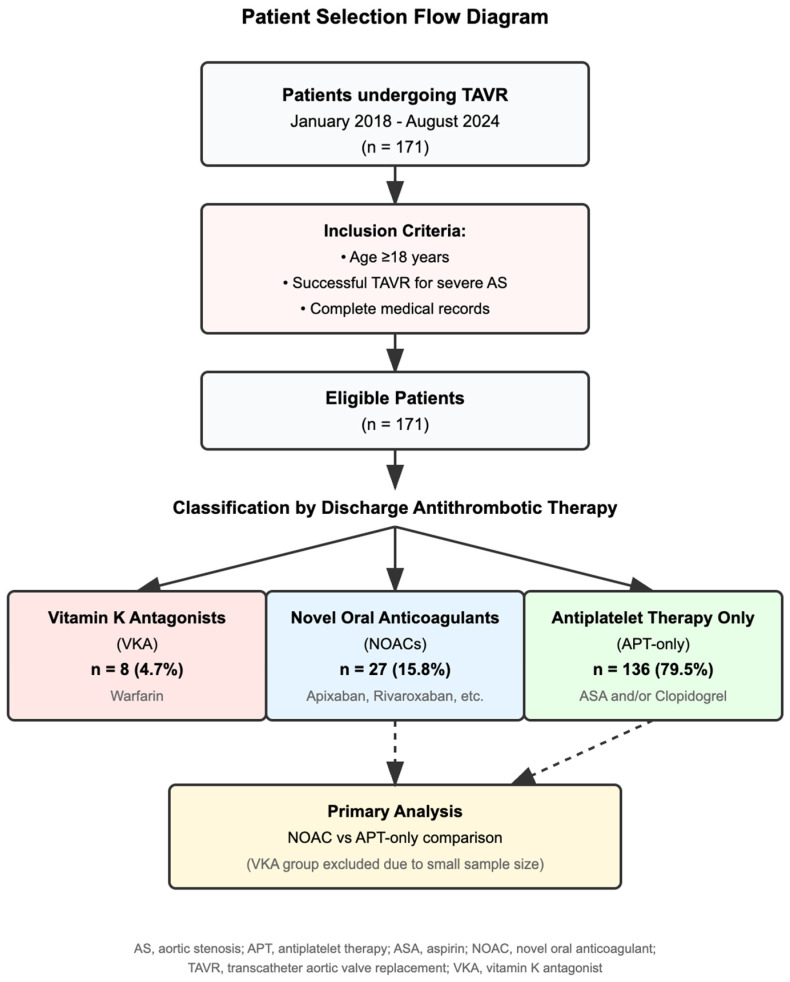
Patient selection flow diagram and distribution of the anticoagulation groups. Legend: Flow diagram showing the selection process of patients who underwent TAVR between January 2018 and August 2024, including the inclusion/exclusion criteria and final distribution among the anticoagulation groups. NOAC, novel oral anticoagulant; TAVR, transcatheter aortic valve replacement; VKA: Vitamin K antagonist.

We conducted a retrospective cohort study of patients who underwent TAVR at Cape Fear Valley Medical Center between January 2018 and August 2024. The study was approved by the Institutional Review Board (IRB 1188-24), which waived the requirement for informed consent. We included adults (≥18 years) who underwent successful TAVR for severe aortic stenosis and excluded those with incomplete records, prior valve surgery, contraindications to anticoagulation, or life expectancy < 1 year from non-cardiac causes (Figure 1).

### 2.2. Antithrombotic Groups

Patients were classified according to the discharge antithrombotic regimen into three groups: (1) novel oral anticoagulants (NOACs)—apixaban, rivaroxaban, edoxaban, or dabigatran; (2) Vitamin K antagonists (VKAs)—warfarin with a target INR of 2.0–3.0; and (3) antiplatelet therapy only (APT-only)—aspirin (75–100 mg), clopidogrel (75 mg), or dual antiplatelet therapy (DAPT) without oral anticoagulation (Table 1 and Table 2). Both anticoagulated groups received concomitant antiplatelet therapy. The APT-only group received standard care for patients without anticoagulation indications. Detailed antithrombotic regimens and indications for anticoagulation were extracted from medical records and categorized as atrial fibrillation, venous thromboembolism, valve thrombosis concern, or other indications.

### 2.3. Outcomes

The primary outcomes were 30-day and 1-year mortality and major adverse cardiovascular and cerebrovascular events (MACCE), defined as all-cause death, myocardial infarction, stroke, or transient ischemic attack. Secondary outcomes included bleeding events according to the Bleeding Academic Research Consortium (BARC) criteria and non-major adverse cardiovascular events (NACE). Data were collected through chart reviews and follow-up encounters. All outcomes are summarized in Table 3, Table 4, Table 5 and Table 6.

### 2.4. Statistical Analysis

The markedly unequal group sizes (NOACs (n = 27); VKAs (n = 8); and APT-only (n = 136) necessitated the modification of our analysis plan. With only eight patients receiving VKA (<5% of the cohort), we had <20% power to detect meaningful differences in VKA. Therefore, we conducted a primary analysis comparing NOACs to APT-only using chi-square tests for categorical variables and Mann−Whitney U tests for continuous variables.

We applied inverse probability of treatment weighting (IPTW) using propensity scores from random forest models to adjust for confounding factors. The variables included in the propensity score model (Table 1) were selected based on clinical relevance and included demographics, comorbidities, procedural characteristics, and baseline risk scores. Binary outcomes were analyzed using logistic regression and time-to-event outcomes using Cox proportional hazards models (Table 6), reporting odds ratios and hazard ratios with 95% confidence intervals.

To assess the validity of our IPTW approach, we evaluated covariate balance using standardized mean differences (SMD) before and after weighting, with SMD < 0.1 indicating adequate balance (Supplementary Table S2). The performance of our propensity score model was assessed using the c-statistic, and the variable importance was extracted from the random forest model to identify the key predictors of treatment assignment. We conducted an overlap assessment by examining the distribution of propensity scores between the groups. The IPTW-adjusted outcomes are presented in Table 5.

### 2.5. Sensitivity and Subgroup Analyses

We performed several sensitivity analyses to assess the robustness of our findings: (1) restricting the analysis to patients with atrial fibrillation as the anticoagulation indication, (2) excluding patients who died within 7 days to account for early procedural complications, (3) stratifying by year of procedure to account for temporal trends (Table 3), and (4) using different IPTW trimming thresholds (1st–99th percentile vs. 5th–95th percentile). Prespecified subgroup analyses examined outcomes stratified by anticoagulation indication, age (≥80 vs. <80 years), CHA_2_DS_2_-VASc score (≥4 vs. <4), and baseline bleeding risk (Supplementary Table S5). “In the sensitivity analysis stratified by the 2020 valve guideline publication, the results remained consistent. For procedures performed during 2021–2024 (post-guideline era), NOACs remained associated with worse outcomes compared to APT-only (1-year mortality HR 2.17, 95% CI 1.13–4.17, *p* = 0.020).”

### 2.6. Multiple Testing Adjustment

Given the multiple outcomes assessed, we applied the Benjamini-Hochberg false discovery rate (FDR) correction with a 5% FDR threshold. Both uncorrected and FDR-adjusted p-values are reported to maintain transparency (Supplementary Table S4).

The VKA group is presented descriptively only, without statistical comparisons (Supplementary Table S1). All analyses were performed using SAS software (version 9.4) with a two-sided α of 0.05. Missing data were minimal (<5%) and were handled using complete case analysis.

## 3. Results

### 3.1. Study Population

Among the 171 patients who underwent TAVR during the study period, 136 (79.5%) received antiplatelet therapy only, 27 (15.8%) received NOACs, and 8 (4.7%) received VKAs (Table 2). The APT-only group consisted predominantly of patients on DAPT (n = 118, 86.8%), with fewer patients receiving aspirin monotherapy (n = 8, 5.9%) or clopidogrel monotherapy (n = 10, 7.4%). Given the small VKA sample size, statistical analyses were restricted to comparing the NOACs and APT-only groups, with VKA outcomes reported descriptively.

### 3.2. Baseline Characteristics

Patients receiving NOACs had higher baseline risk profiles than those in the APT-only group (Table 4), including higher mean CHA_2_DS_2_-VASc scores (4.7 ± 2.0 vs. 2.0 ± 2.5, *p* < 0.001) and more frequent indications for anticoagulation (93% vs. 13%, *p* < 0.001), predominantly atrial fibrillation. Patients receiving NOACs more often had prior cerebrovascular events (41% vs. 10%, *p* < 0.001) and higher STS-PROM scores (7.9 ± 8.8 vs. 6.0 ± 8.9, *p* = 0.056). The groups were similar in terms of age (76.4 ± 8.0 vs. 76.3 ± 8.8 years, *p* = 0.225), sex distribution, and prevalence of coronary artery disease.

### 3.3. Clinical Outcomes: NOAC Versus APT-Only

At 30 days, NOAC patients had significantly lower survival than APT-only (67% vs. 88%, *p* = 0.017), translating to an unadjusted odds ratio of 0.27 (95% CI 0.10–0.69, *p* = 0.007) (Table 4). The incidence of MACCE was higher in the NOAC group (22% vs. 8%, *p* = 0.051), with an odds ratio of 3.25 (95% CI 1.08–9.72, *p* = 0.035) (Table 6). Bleeding events were uncommon and similar between the groups (7% vs. 4%, *p* = 0.649).

**Figure 2:**
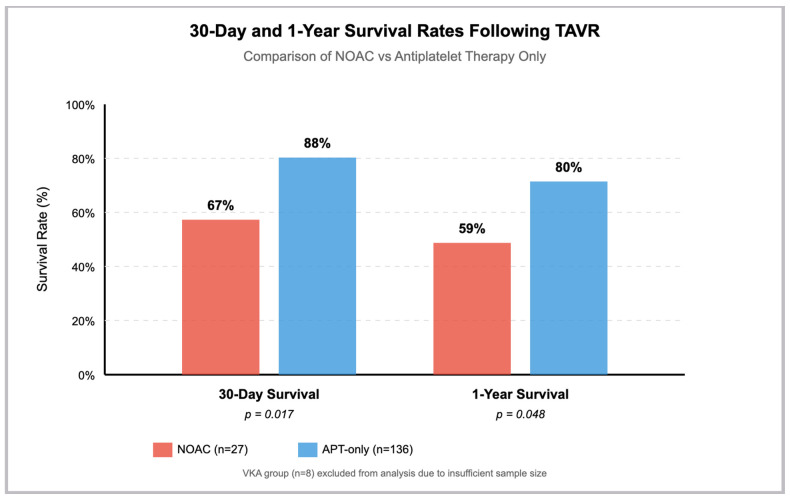
Post-TAVR survival: NOAC versus antiplatelet therapy Survival rates at 30 days and 1 year post-TAVR for NOAC (n = 27, red) and APT-only (n = 136, blue) groups. Patients receiving NOAC showed significantly lower survival rates at both timepoints (*p* = 0.017 and *p* = 0.048, respectively). The VKA group (n = 8) was excluded due to insufficient sample size.

These differences persisted at 1 year, with NOAC patients showing lower survival (59% vs. 80%, *p* = 0.048) and higher MACCE rates (34% vs. 17%, *p* < 0.001). After IPTW adjustment for baseline differences (Table 5), NOACs remained associated with worse outcomes: 30-day MACCE odds ratio 5.59 (95% CI 2.56–12.18, *p* < 0.001), 1-year mortality hazard ratio 2.22 (95% CI 1.22–4.03, *p* = 0.009), and 1-year MACCE odds ratio 2.40 (95% CI 1.23–4.68, *p* = 0.010) (Table 6) (Figure 2).

### 3.4. Temporal Trends

The proportion of patients receiving NOACs increased over the study period, from 4% in 2019 to 30% in 2023–2024 (rank-biserial correlation, *p* = 0.003), while APT-only use correspondingly decreased (Table 3). This trend likely reflects evolving practice patterns favoring NOACs over VKAs when anticoagulation is indicated.

### 3.5. Descriptive VKA Outcomes

The eight patients receiving VKAs had a mean age of 80.6 ± 7.9 years, 50% were female, and all had indications for anticoagulation (Supplementary Table S1). Seven of the eight patients (87.5%) survived at 30 days and 1 year. No patients treated with VKA experienced MACCE or bleeding events at either time point. While these outcomes appear favorable, no statistical comparisons were performed due to the small sample size.

## 4. Discussion

In this retrospective study of 171 post-TAVR patients, NOACs were associated with significantly worse outcomes than antiplatelet therapy alone, including higher mortality and MACCE rates at both 30 days and 1 year. These associations persisted after propensity score adjustments and sensitivity analyses. The severe underrepresentation of patients receiving VKA (n = 8, <5%) precluded any meaningful NOAC-VKA comparison, fundamentally limiting our ability to address the original research question of comparing NOACs with VKAs in post-TAVR patients.

### 4.1. Key Findings and Context

Patients prescribed NOACs exhibited shorter survival times at both the 30-day and 1-year follow-ups, as well as lower overall survival from baseline. Additionally, these patients experienced MACCE and NACE more frequently than those without. The use of inverse probability of treatment weighting enhanced some of the differences between groups or amplified those that were statistically significant.

Our results align with the GALILEO trial, which was terminated early due to excess death and bleeding with rivaroxaban [5], but contrast with ATLANTIS, which showed similar outcomes between apixaban and standard care [7]. This study is particularly relevant given recent research in other populations. Overtchouk et al. found that NOACs are associated with reduced major bleeding risk and lower mortality rates in patients with atrial fibrillation [8], while Brouwer et al. indicated that NOACs might provide comparable or even favorable outcomes regarding cardiovascular events in other high-risk procedures [9]. However, our findings suggest these benefits may not extend to the post-TAVR population.

Adverse NOAC outcomes are particularly concerning, given that 87% of our APT-only group received DAPT, an active antithrombotic strategy. The consistency of the findings across patients with and without atrial fibrillation suggests that the risk is not limited to specific subgroups.

Our findings should be interpreted in the context of evolving TAVR antithrombotic practices; most modern valve centers have moved away from VKAs post-TAVR unless there is another strong indication, with current practice favoring DAPT initially, followed by aspirin monotherapy after 6 months. The small VKA group in our study likely reflects this temporal shift in practice patterns, with VKAs primarily used in the early TAVR era.

### 4.2. Methodological Considerations

Our IPTW approach balanced the measured covariates (all SMDs < 0.1 post-weighting) with good model discrimination (c-statistic 0.842). Key findings remained significant after FDR correction, particularly for MACCE outcomes. However, residual confounding from unmeasured factors likely persists, as NOAC patients’ higher baseline risk despite adjustment suggests unmeasured characteristics influencing treatment selection.

Nearly 80% of patients belonged to the antiplatelet therapy-only group, while only eight patients (slightly less than 5%) were in the VKA group, which constrained many analyses involving this group. The use of NOACs demonstrated an increasing trend over the study period, with a higher proportion of patients being prescribed these medications in recent years (4% in 2019 to 30% in 2023–2024).

### 4.3. Limitations

The main limitations of this study include its retrospective nature, single-center design, and small sample size, particularly in the VKA group. These factors limit the generalizability of the results and highlight the need for larger prospective studies. We lacked data on specific NOAC agents, dosing, timing of initiation, and validated bleeding-risk scores. The safety-net hospital setting may not reflect the outcomes of high-volume TAVR centers.

## 5. Conclusions

In this single-center retrospective study, NOACs were associated with increased mortality and MACCE compared with antiplatelet therapy alone in post-TAVR patients. The extremely limited VKA representation (n = 8) precluded any meaningful NOAC-VKA comparison, fundamentally altering our ability to address the research question. These findings suggest that caution should be exercised when considering NOACs for post-TAVR patients without clear indications for anticoagulation. Prospective randomized trials with adequate sample sizes across all antithrombotic strategies are urgently needed to guide clinical decision-making.

## Figures and Tables

**Table 1:** Variables tested using a set of random forest regression models to predict anticoagulant type.

Year of TAVR procedure
Sex
Weight
Height
CHA_2_DS_2_-VASc score
Indication for oral anticoagulant therapy
Chronic obstructive lung disease
History of prior cerebrovascular event, myocardial infarction, coronary bypass graft, percutaneous coronary intervention
Chronic kidney disease
NYHA functional class
Malignancy
Hemoglobin level at admission
Multivessel coronary artery disease
Left ventricular ejection fraction
Aortic valve area
TAVR in the bioprosthesis procedure
Implanted prosthesis size
Pre-dilation performed
Major VARC-2 vascular complications
Acute kidney injury Stage II or III
Antithrombotic therapy at discharge

NYHA: New York Heart Association; TAVR: Transcatheter Aortic Valve Replacement; VARC-2: Valve Academic Research Consortium-2 initiative.

**Table 2:** Distribution of antithrombotic therapy groups.

Antithrombotic Strategy	n	%	Included in Primary Analysis
Antiplatelet therapy only	136	79.5%	Yes
- Aspirin monotherapy	8	5.9% *	
- Clopidogrel monotherapy	10	7.4% *	
- DAPT	118	86.8% *	
NOACs	27	15.8%	Yes
VKAs	8	4.7%	No (descriptive only)
Total	171	100%	

* Percentage of the APT-only group.

**Table 3:** Primary analysis—NOAC vs. APT-only outcomes.

Outcome	NOACs (n = 27)	APT-Only (n = 136)	Unadjusted		IPTW-Adjusted	
	n (%)	n (%)	OR/HR (95% CI)	*p*	OR/HR (95% CI)	*p*
30-day						
Survival	18 (67%)	120 (88%)	0.27 (0.10–0.69)	0.007	0.35 (0.17–0.72)	0.004
MACCE	6 (22%)	11 (8%)	3.25 (1.08–9.72)	0.035	5.59 (2.56–12.18)	<0.001
1-year						
Survival	16 (59%)	109 (80%)	0.36 (0.15–0.87)	0.022	0.47 (0.25–0.90)	0.022
MACCE	9 (34%)	22 (17%)	2.58 (1.02–6.53)	0.045	2.40 (1.23–4.68)	0.010

**Table 4:** Descriptive statistics of the study variables.

	Non-VKAs (n = 27)	No Therapy, ASA, or Clopidogrel (n = 136)	All (N = 163)	*p* Value
Age	76.41 ± 8.04	76.28 ± 8.83	76.5 ± 8.67	0.225
Women	8 (30%)	72 (47%)	87 (51%)	0.086
Weight	95.59 ± 25.01	87.11 ± 23.14	88.08 ± 23.31	0.195
Height	1.73 ± 0.11	1.66 ± 0.12	1.67 ± 0.12	0.055
STS-PROM	7.87 ± 8.75	6.02 ± 8.85	6.44 ± 8.66	0.056
CHA_2_DS_2_-VASc score	4.7 ± 2.00	2.03 ± 2.46	2.6 ± 2.61	*p* < 0.001
Indication for oral anticoagulant therapy (any)	25 (93%)	18 (13%)	43 (30%)	*p* < 0.001
Permanent atrial fibrillation	12 (44%)	9 (7%)	21 (14%)	*p* < 0.001
Paroxysmal atrial fibrillation	13 (48%)	8 (6%)	21 (15%)	
Others	0 (0%)	1 (1%)	1 (1%)	
Hypertension	27 (100%)	128 (94%)	155 (95%)	0.340
Diabetes mellitus	16 (59%)	72 (53%)	88 (54%)	0.746
Peripheral artery disease	11 (41%)	65 (48%)	76(46%)	0.702
Chronic obstructive lung disease	10 (38%)	30 (22%)	40 (25%)	0.186
History of cerebrovascular event	11 (41%)	13 (10%)	24 (15%)	*p* < 0.001
History of myocardial infarction	9 (33%)	20 (15%)	29 (18%)	0.063
History of aortocoronary bypass graft surgery	2 (8%)	5 (10%)	7 (11%)	0.047
History of percutaneous coronary intervention	8 (30%)	50 (37%)	58 (37%)	0.238
Chronic kidney disease	15 (56%)	72 (53%)	82 (53%)	0.953
NYHA functional class (mean rank comparison)	2.78 ± 0.75	2.43 ± 0.92	2.51 ± 0.90	0.061
Class I	2 (7%)	29 (21%)	32 (18%)	0.270
Class II	5 (19%)	31 (23%)	36 (22%)	
Class III	17 (63%)	65 (48%)	82 (50%)	
Class IV	3 (11%)	11 (8%)	14 (9%)	
Malignancy	5 (19%)	37 (27%)	42 (26%)	0.640
Hemoglobin at admission	12.18 ± 2.06	11.74 ± 1.92	11.85 ± 1.94	0.255
Congestive heart failure	15 (56%)	56 (41%)	71 (45%)	0.085
Multivessel coronary artery disease	14 (52%)	64 (47%)	78 (48%)	0.895
Left ventricular ejection fraction	51.56 ± 15.37	53.31 ± 13.32	52.78 ± 13.65	0.217
Aortic valve area	0.7 ± 0.25	0.7 ± 0.22	0.69 ± 0.23	0.571
TAVR in bioprosthesis	22 (82%)	83 (61%)	45 (66%)	0.014
Implanted prosthesis size	25.85 (2.74)	24.71 (2.26)	24.95 (2.40)	0.054
Pre-dilation performed	10 (37%)	92 (68%)	104 (61%)	0.001
Major VARC-2 vascular complications	2 (7%)	3 (2%)	5 (3%)	0.301
Acute kidney injury stage II or III	3 (11%)	5 (4%)	8 (5%)	0.201
Antithrombotic therapy at discharge				*p* < 0.001
Aspirin	2 (7%)	8 (6%)	10 (6%)	
ADP receptor inhibitors alone	19 (70%)	10 (7%)	29 (20%)	
DAPT	6 (22%)	118 (87%)	124 (74%)	
30-days survival	18 (67%)	120 (88%)	138 (84%)	0.017
30-days, any BARC bleeding	2 (7%)	6 (4%)	8(5%)	0.649
No therapy, BARC 0	25 (93%)	130 (96%)	155 (95%)	0.712
Minor, BARC 1	2 (7%)	4 (3%)	6 (4%)	
Major or life-threatening, BARC 2	0 (0%)	2 (2%)	2 (1%)	
30 days, MACCE	6 (22%)	11 (8%)	17 (10%)	0.051
30 days, NACE	6 (22%)	17 (13%)	23 (14%)	0.410
1-year survival	16 (59%)	109 (80%)	125 (77%)	0.048
1-year cardio-cerebrovascular mortality	1 (4%)	8 (6%)	9 (5%)	0.712
1-year, any BARC bleeding	4 (15%)	14 (10%)	18 (11%)	0.478
No therapy, BARC 0	23 (85%)	122 (90%)	143 (90%)	0.677
Minor, BARC 1	2 (7%)	4 (3%)	6 (4%)	
Major or life-threatening, BARC 2	2 (7%)	10 (7%)	12 (7%)	
1 year, Cerebrovascular events				0.904
No therapy	27 (100%)	128 (94%)	163 (95%)	
Transient ischemic event	0 (0%)	4 (3%)	4 (2%)	
Nondisabling	0 (0%)	2 (2%)	2 (1%)	
Disabling	0 (0%)	2 (2%)	2 (1%)	
1 year, MACCE	7 (26%)	22 (16%)	29 (17%)	0.198
1 year, NACE	8 (30%)	32 (24%)	41 (24%)	0.587
Survival time (of those registered to have died), in days	72 ± 104	390 ± 511	320 ± 461	0.055
Survival time merged, in days *	642 ± 571	956 ± 575	892 ± 578	0.021

* With alive patients calculated until 1 August 2024. Note. Values in the table are either frequencies (% of patients within an anticoagulant category) or mean ± standard deviation. *p*-values for variables represented by frequencies and percentages are chi-square *p*-values. For those represented as mean ± standard deviation, *p*-values are Kruskal−Wallis test results. ASA: aspirin; ADP: adenosine diphosphate; BARC: Bleeding Academic Research Consortium; DAPT: dual antiplatelet therapy; MACCE: major adverse cardiovascular events; NACE: non-major adverse cardiovascular events; NYHA: New York Heart Association; STS-PROM: Society of Thoracic Surgeons Predicted Risk of Mortality; TAVR: Transcatheter Aortic Valve Replacement; VARC-2: Valve Academic Research Consortium-2 initiative; VKA: Vitamin K antagonist.

**Table 5:** Inverse probability of treatment weighting adjusted the values of the outcome variables.

	Non-VKAs (n = 62)	ATP (n = 156)	All (N = 181)	*p* Value
30-day survival	44 (71%)	137 (88%)	181 (85%)	0.002
30-days, any BARC bleeding	4 (7%)	7 (5%)	11 (4%)	0.254
No therapy, BARC 0	58 (94%)	149 (96%)	251 (96%)	0.325
Minor, BARC 1	4 (7%)	5 (3%)	9 (3%)	
Major or life-threatening, BARC 2	0 (0%)	2 (1%)	2 (1%)	
30 days, MACCE	21 (34%)	13 (8%)	34 (13%)	*p* < 0.001
30 days, NACE	20 (32%)	19 (12%)	39 (17%)	0.002
1-year survival	40 (65%)	124 (80%)	164 (78%)	0.002
1-year cardio-cerebrovascular mortality	3 (5%)	9 (6%)	12 (5%)	0.269
1-year, any BARC bleeding	8 (13%)	17 (11%)	25 (10%)	0.056
No therapy, BARC 0	54 (87%)	140 (90%)	194 (91%)	0.042
Minor, BARC 1	5 (8%)	4 (3%)	9 (3%)	
Major or life-threatening, BARC 2	3 (5%)	12 (8%)	15 (6%)	
1 year, cerebrovascular events				0.315
No therapy	62 (100%)	146 (94%)	208 (96%)	
Transient ischemic event	0 (0%)	5 (3%)	5 (2%)	
Nondisabling	0 (0%)	2 (1%)	2 (1%)	
Disabling	0 (0%)	3 (2%)	3 (1%)	
1 year, MACCE	21 (34%)	27 (17%)	48 (18%)	*p* < 0.001
1 year, NACE	23 (37%)	39 (25%)	68 (26%)	0.031
Survival time, in days *	65 ± 87	376 ± 493	283 ± 409	0.002
Survival time merged ^†^	679 ± 550	937 ± 576	870 ± 563	0.033

* Of those registered to have died. ^†^ With alive patients calculated until 1 August 2024. Note. Values in the table are either frequencies (% of weights within an anticoagulant category) or mean ± standard deviation. *p*-values for variables represented by frequencies and percentages are chi-square *p*-values. For those represented as mean ± standard deviation, *p*-values are Kruskal−Wallis test results. where n is the sum of the weights in the category. BARC: Bleeding Academic Research Consortium; MACCE: major adverse cardiovascular events; NACE: non-major adverse cardiovascular events; VKA: Vitamin K antagonist ATP anitplatelet therapy.

**Table 6:** Cox regression analysis predicting mortality based on study group binary variables.

Cox Regression	Unadjusted		Adjusted, Using Inverse Probability of Treatment Weights	
Group compared with no therapy	Non-VKA oral anticoagulants		Non-VKA oral anticoagulants	
	HR (95% CI)	*p* value	HR (95% CI)	*p* value
Overall mortality (survival time merged)	1.737 (0.886–3.408)	0.108	1.440 (0.854–2.427)	0.171
Overall mortality (survival since baseline)	2.057 (0.996–4.250)	0.051	2.219 (1.222–4.026)	0.009

Note. Each column presents the results of a Cox regression in which a binary variable contained data on whether the patient was in the no-therapy group or in the listed anticoagulant group. Each procedure was based on a comparison of two groups, one of which was the no-therapy group, and the other was listed in the column. A hazard ratio above 1 indicates that the risk of death in the studied period in a unit of time is higher in the listed anticoagulant group, and hazard ratios below 1 indicate that the risk of death in a unit of time is higher in the no-therapy group. CI: confidence interval; HR: hazard ratio; VKA: Vitamin K antagonist.

## Data Availability

The data that support the findings of this study are available from the corresponding author, Ricardo A. Rodriguez Mejia, upon reasonable request. The data are not publicly available because they contain information that could compromise the privacy of the research participants. Requests for access to the dataset used and analyzed in the current study will be reviewed by the Cape Fear Valley Health System Institutional Review Board and are subject to approval. Any data sharing will be restricted to non-identifiable data due to the nature of the study and to protect patient privacy. The statistical code used for the analysis is available from the corresponding author without undue reservation. Any shared data will be provided in accordance with the consent provided by participants on the use of confidential data and adherence to HIPAA regulations.

## Supplementary Materials

The following supporting information can be downloaded at: https://www.mdpi.com/article/10.3390/jcm14134690/s1, Table S1: Descriptive Characteristics and Outcomes of VKA Group (n = 8) *; Table S2: Standardized Mean Differences Pre- and Post-IPTW for NOAC vs APT-only Comparison; Table S3: Outcomes Stratified by Anticoagulation Indication; Table S4: False Discovery Rate Correction for Primary and Secondary Outcomes; Table S5: Sensitivity Analyses for Primary Outcomes.

## Permission to Reproduce Material from Other Sources

The following research includes citations and references to previously published works, which have been properly documented according to academic standards. All cited works are duly acknowledged through in-text citations and complete bibliographic references, in accordance with scholarly conventions. These citations serve to recognize the intellectual contributions of other researchers and support the arguments and findings presented in this study.
