# Supplementary material for: Novel Oral Anticoagulants Versus Antiplatelet Therapy in Post-TAVR Patients: A Single-Center Retrospective Study
Source: J Clin Med. 2025 Jul 2;14(13):4690. doi: 10.3390/jcm14134690 (PMC12250888; doi:10.3390/jcm14134690)
Supplement: Supplementary file 1 [file jcm-14-04690-s001.zip › jcm-3705282-supplementary.pdf]

Supplementary data

Supplementary Table S1: Descriptive Characteristics and Outcomes of VKA Group (n=8)*

Characteristic	Value
**Demographics**	
Age, mean ± SD	80.6 ± 7.9
Female sex, n (%)	4 (50.0)
BMI, mean ± SD	27.3 ± 5.2
**Clinical Characteristics**	
STS-PROM score, mean ± SD	7.9 ± 4.6
CHA_2DS_2-VASc score, mean ± SD	5.1 ± 1.6
NYHA Class III-IV, n (%)	6 (75.0)
LVEF, mean ± SD	48.0 ± 13.9
**Anticoagulation Indication**	
Atrial fibrillation, n (%)	8 (100.0)
- Permanent	3 (37.5)
- Paroxysmal	5 (62.5)
**Comorbidities**	
Hypertension, n (%)	8 (100.0)
Diabetes mellitus, n (%)	5 (62.5)
Prior CVA/TIA, n (%)	2 (25.0)
Prior CABG, n (%)	3 (37.5)
CKD, n (%)	4 (50.0)
**Procedural Characteristics**	
Bioprosthetic valve, n (%)	8 (100.0)
Valve size, mean ± SD	26.0 ± 2.8
Pre-dilation performed, n (%)	2 (25.0)
**Antithrombotic at Discharge**	
VKA + SAPT, n (%)	5 (62.5)
VKA + DAPT, n (%)	3 (37.5)
**Clinical Outcomes**	
30-day survival, n (%)	7 (87.5)
30-day MACCE, n (%)	0 (0.0)
30-day bleeding (any BARC), n (%)	0 (0.0)
1-year survival, n (%)	7 (87.5)
1-year MACCE, n (%)	0 (0.0)
1-year bleeding (any BARC), n (%)	0 (0.0)

*Note: No statistical comparisons performed due to small sample size. Data presented for descriptive purposes only.

Abbreviations: BARC, Bleeding Academic Research Consortium; BMI, body mass index; CABG, coronary artery bypass graft; CKD, chronic kidney disease; CVA, cerebrovascular accident; DAPT, dual antiplatelet therapy; LVEF, left ventricular ejection fraction; MACCE, major adverse

cardiovascular and cerebrovascular events; NYHA, New York Heart Association; SAPT, single antiplatelet therapy; STS-PROM, Society of Thoracic Surgeons Predicted Risk of Mortality; TIA, transient ischemic attack; VKA, vitamin K antagonist

Supplementary Table S2: Standardized Mean Differences Pre- and Post-IPTW for NOAC vs APT-only Comparison

Variable	Pre-IPTW SMD	Post-IPTW SMD	Balance Achieved*
**Demographics**			
Age	0.012	0.003	✓
Female sex	0.226	0.052	✓
BMI	0.185	0.041	✓
**Risk Scores**			
CHA_2DS_2-VASc score	0.684	0.078	✓
STS-PROM score	0.201	0.045	✓
**Anticoagulation Indication**			
Any indication	1.423	0.092	✓
Atrial fibrillation	1.387	0.089	✓
**Comorbidities**			
Hypertension	0.162	0.038	✓
Diabetes mellitus	0.127	0.029	✓
Prior CVA/TIA	0.743	0.064	✓
Prior MI	0.412	0.055	✓
Prior CABG	-0.098	0.022	✓
Prior PCI	-0.156	0.035	✓
CKD	0.056	0.013	✓
COPD	0.348	0.048	✓
CHF	0.312	0.042	✓
PAD	-0.147	0.033	✓
**Clinical Parameters**			
NYHA Class III-IV	0.285	0.039	✓
LVEF	-0.124	0.028	✓
Aortic valve area	0.000	0.000	✓
Hemoglobin	0.216	0.049	✓
**Procedural Factors**			
Bioprosthetic valve	0.456	0.061	✓
Valve size	0.498	0.067	✓
Pre-dilation	-0.684	-0.077	✓
Year of procedure	0.312	0.044	✓

*Balance achieved defined as SMD <0.1

Model Performance Metrics:

- C-statistic for propensity score model: 0.842 (95% CI: 0.761-0.923)

- Effective sample size after IPTW: NOAC = 24.3, APT-only = 127.8
- Maximum weight: 3.21
- Mean weight: 1.00 (by design)

Abbreviations: APT, antiplatelet therapy; BMI, body mass index; CABG, coronary artery bypass graft; CHF, congestive heart failure; CKD, chronic kidney disease; COPD, chronic obstructive pulmonary disease; CVA, cerebrovascular accident; IPTW, inverse probability of treatment weighting; LVEF, left ventricular ejection fraction; MI, myocardial infarction; NOAC, novel oral anticoagulant; NYHA, New York Heart Association; PAD, peripheral artery disease; PCI, percutaneous coronary intervention; SMD, standardized mean difference; STS-PROM, Society of Thoracic Surgeons Predicted Risk of Mortality; TIA, transient ischemic attack

Supplementary Table S3: Outcomes Stratified by Anticoagulation Indication

Subgroup	Treatment	N	30-Day Survival n (%)	1-Year Survival n (%)	30-Day MACCE n (%)	1-Year MACCE n (%)
**Atrial Fibrillation**						
	NOAC	22	15 (68.2)	13 (59.1)	5 (22.7)	8 (36.4)
	APT-only	18	16 (88.9)	14 (77.8)	2 (11.1)	3 (16.7)
	p-value*		0.226	0.213	0.414	0.273
**No Atrial Fibrillation**						
	NOAC	5	3 (60.0)	3 (60.0)	1 (20.0)	1 (20.0)
	APT-only	118	104 (88.1)	95 (80.5)	9 (7.6)	19 (16.1)
	p-value*		0.128	0.302	0.346	1.000
**Other Anticoagulation Indications†**						
	NOAC	5	3 (60.0)	3 (60.0)	1 (20.0)	1 (20.0)
	VTE	3	2 (66.7)	2 (66.7)	1 (33.3)	1 (33.3)
	Valve concern	2	1 (50.0)	1 (50.0)	0 (0.0)	0 (0.0)

*P-values from Fisher's exact test for categorical comparisons. Interpret with caution due to small subgroup sizes. †Detailed breakdown provided for descriptive purposes only; no statistical comparisons performed due to very small numbers.

Hazard Ratios for Mortality (NOAC vs APT-only):

- Atrial fibrillation subgroup: HR 2.84 (95% CI: 1.23-6.55), p=0.014
- No atrial fibrillation subgroup: HR 1.98 (95% CI: 0.58-6.73), p=0.274
- Interaction p-value: 0.621

Note: Subgroup analyses are exploratory and underpowered. Results should be considered hypothesis-generating only.

Abbreviations: APT, antiplatelet therapy; CI, confidence interval; HR, hazard ratio; MACCE, major adverse cardiovascular and cerebrovascular events; NOAC, novel oral anticoagulant; VTE, venous thromboembolism.

Supplementary Table S4: False Discovery Rate Correction for Primary and Secondary Outcomes

Outcome	Comparison	Uncorrected p-value	FDR-adjusted p-value*	Remains Significant†
**Primary Outcomes**				
30-day mortality	NOAC vs APT-only	0.017	0.051	No
30-day MACCE	NOAC vs APT-only	<0.001	0.002	Yes
1-year mortality	NOAC vs APT-only	0.048	0.072	No
1-year MACCE	NOAC vs APT-only	<0.001	0.001	Yes
**Secondary Outcomes**				
30-day any bleeding	NOAC vs APT-only	0.649	0.721	No
30-day NACE	NOAC vs APT-only	0.410	0.513	No
1-year any bleeding	NOAC vs APT-only	0.478	0.574	No
1-year CV mortality	NOAC vs APT-only	0.654	0.721	No
1-year NACE	NOAC vs APT-only	0.502	0.574	No
**IPTW-Adjusted Analyses**				
30-day mortality (OR)	NOAC vs APT-only	0.004	0.016	Yes
30-day MACCE (OR)	NOAC vs APT-only	<0.001	0.002	Yes
1-year mortality (HR)	NOAC vs APT-only	0.009	0.018	Yes
1-year MACCE (OR)	NOAC vs APT-only	0.010	0.020	Yes

*Benjamini-Hochberg false discovery rate correction applied with FDR threshold of 0.05
†Significant if FDR-adjusted p-value <0.05

Summary:

- Number of tests performed: 13
- Number remaining significant after FDR correction: 6
- Critical p-value threshold (Benjamini-Hochberg): 0.023

Note: FDR correction helps control the expected proportion of false discoveries among rejected hypotheses. The most robust findings (30-day and 1-year MACCE) remain significant after correction.

Abbreviations: APT, antiplatelet therapy; CV, cardiovascular; FDR, false discovery rate; HR, hazard ratio; IPTW, inverse probability of treatment weighting; MACCE, major adverse cardiovascular and cerebrovascular events; NACE, non-major adverse cardiovascular events; NOAC, novel oral anticoagulant; OR, odds ratio

Supplementary Table S5: Sensitivity Analyses for Primary Outcomes

Analysis	N (NOAC/APT)	1-Year Mortality HR (95% CI)	p-value	1-Year MACCE OR (95% CI)	p-value
**Primary Analysis**	27/136	2.22 (1.22-4.03)	0.009	2.40 (1.23-4.68)	0.010
**Sensitivity Analyses**					
1. AF patients only	22/18	2.84 (1.23-6.55)	0.014	2.88 (1.15-7.22)	0.024
2. Excluding early deaths*	25/131	2.15 (1.14-4.05)	0.018	2.35 (1.18-4.69)	0.015
3. IPTW trim 1st-99th %ile	27/136	2.18 (1.19-3.99)	0.011	2.42 (1.24-4.73)	0.009
4. IPTW trim 5th-95th %ile	26/133	2.09 (1.13-3.87)	0.019	2.31 (1.16-4.60)	0.017
**By Procedure Year**					
2018-2020	5/54	2.41 (0.71-8.19)	0.157	2.65 (0.68-10.31)	0.159
2021-2024	22/82	2.17 (1.13-4.17)	0.020	2.33 (1.14-4.77)	0.021
**By Age Group**					
Age <80 years	16/78	2.35 (1.05-5.26)	0.037	2.51 (1.02-6.18)	0.045
Age ⩾80 years	11/58	2.08 (0.91-4.75)	0.082	2.28 (0.93-5.59)	0.071
**By CHA_2DS_2-VASc Score**					
Score <4	10/95	1.89 (0.68-5.25)	0.221	2.12 (0.71-6.33)	0.177
Score ⩾4	17/41	2.46 (1.19-5.08)	0.015	2.58 (1.18-5.64)	0.017
**By Bleeding Risk†**					
Low bleeding risk	19/92	2.31 (1.12-4.76)	0.023	2.48 (1.14-5.39)	0.022
High bleeding risk	8/44	2.05 (0.78-5.39)	0.146	2.21 (0.77-6.35)	0.139

*Excluding patients who died within 7 days of TAVR †High bleeding risk defined as prior major bleeding, CKD stage ≥4, or concurrent DAPT requirement

Key Findings:

- Primary results remain robust across most sensitivity analyses
- Effect appears consistent across temporal periods despite changing practice patterns
- Higher risk patients (CHA_2DS_2-VASc ⩾4) show stronger association
- Results consistent with different IPTW trimming approaches

Note: Some subgroup analyses have limited power due to small sample sizes. Interpret with caution.

Abbreviations: AF, atrial fibrillation; APT, antiplatelet therapy; CI, confidence interval; CKD, chronic kidney disease; DAPT, dual antiplatelet therapy; HR, hazard ratio; IPTW, inverse probability of treatment weighting; MACCE, major adverse cardiovascular and

cerebrovascular events; NOAC, novel oral anticoagulant; OR, odds ratio; TAVR, transcatheter aortic valve replacement
